# *Candida albicans* Antifungal Resistance and Tolerance in Bloodstream Infections: The Triad Yeast-Host-Antifungal

**DOI:** 10.3390/microorganisms8020154

**Published:** 2020-01-22

**Authors:** Sofia Costa-de-Oliveira, Acácio G. Rodrigues

**Affiliations:** 1 Division of Microbiology, Department of Pathology, Faculty of Medicine, University of Porto, Al. Hernâni Monteiro, 4200-319 Porto, Portugal; agr@med.up.pt; 2 Center for Research in Health Technologies and Information Systems (CINTESIS), R. Dr. Plácido da Costa, 4200-450 Porto, Portugal; 3 Burn Unit, São João Hospital Center, Al. Hernâni Monteiro, 4200-319 Porto, Portugal

**Keywords:** *C. albicans*, antifungal resistance, bloodstream infections, *Candida* infections, virulence

## Abstract

*Candida albicans* represents the most frequent isolated yeast from bloodstream infections. Despite the remarkable progress in diagnostic and therapeutic approaches, these infections continue to be a critical challenge in intensive care units worldwide. The economic cost of bloodstream fungal infections and its associated mortality, especially in debilitated patients, remains unacceptably high. *Candida albicans* is a highly adaptable microorganism, being able to develop resistance following prolonged exposure to antifungals. Formation of biofilms, which diminish the accessibility of the antifungal, selection of spontaneous mutations that increase expression or decreased susceptibility of the target, altered chromosome abnormalities, overexpression of multidrug efflux pumps and the ability to escape host immune defenses are some of the factors that can contribute to antifungal tolerance and resistance. The knowledge of the antifungal resistance mechanisms can allow the design of alternative therapeutically options in order to modulate or revert the resistance. We have focused this review on the main factors that are involved in antifungal resistance and tolerance in patients with *C. albicans* bloodstream infections.

## 1. Introduction

*Candida albicans* coexists in humans as commensal without damage to the host, colonizing several body locations like the skin, genital tract, and gastro-intestinal tract [1]. Nevertheless, as an opportunistic pathogen, whenever the immune status of the host or its microbiota becomes disturbed, it can cause extensive mucosal colonization and local and/or systemic disease [2,3]. Along the years, in parallel to the advance of medical procedures, the incidence of bloodstream *Candida* infections increased as well as the associated mortality rate, being *C. albicans* the most frequent yeast isolated from patient biological samples [4,5,6,7,8,9].

The intensive care unit (ICU) setting provides *C. albicans* the opportunity for development of infection. Colonization of the skin and mucous membranes and the alteration or disruption of natural host barriers, like wounds, surgery, and the insertion of indwelling intravascular catheters are the main predisposing factors for *Candida* infections [6,10,11].

One of the main factors that contribute to the high mortality rate associated with *Candidaemia* is the difficulty in diagnosis, due to the nonspecific clinical symptoms of systemic fungal infection and delayed laboratorial detection methods, as well as the subsequent delay in initiation of adequate antifungal therapy [12,13]. Unlike antibacterial drugs, the array of available antifungals is somewhat scarcer. Azoles, polyenes, and echinocandins are the three main antifungal classes, being the last considered first-line therapy in many hospitals for the treatment of invasive candidiasis [14,15,16,17].

With the increase of clinical and/or microbiological antifungal resistance or tolerance, susceptibility tests play an ever-increasing role in the selection of antifungal drugs. Notably, correlation between in vitro susceptibility and treatment success is not always straightforward [18]. The in vivo conditions are significantly different of in vitro, in particular the microorganisms are often under the effect of both antifungal and non-antifungal drugs, as is the typical case of critical care patients [19,20,21]. Besides, *C. albicans* has particular traits and tricks that makes this yeast a true challenge for clinicians and researchers.

This review highlights the multiple attributes of *C. albicans* that may influence and promote antifungal resistance and tolerance.

## 2. Antifungal Drugs: Mechanisms of Action and Resistance

The battery of clinical antifungal agents available is somewhat limited, in contrast to antibacterial drugs. They arise from the number of drug targets in fungi, and its similarity to human eukaryotic cells. Nevertheless, pursuit for new cell targets, within the genomic era, has increased exponentially. Patients under long term antifungal prophylaxis or antifungal treatment display favorable conditions for the emergence of antifungal resistance [22].

Three types of antifungal resistance have been described: primary or intrinsic, exhibited before antifungal exposure, secondary or acquired, and clinical resistance [23]. Secondary or acquired resistance develops following exposure to an antifungal agent and can be either reversible, due to transient adaptation, or persistent because of one or several genetic alterations [23].

The main antifungals used in invasive candidiasis treatment, as well as the main mechanisms of action and resistance are summarized in Table 1 and detailed in the next subsections. Clinical resistance will be address in Section 3.

### 2.1. Polyenes

Polyenes belong to a class of natural compounds with a heterocyclic amphipathic molecule (one hydrophilic charged side of the molecule and one hydrophobic, uncharged side). They target ergosterol in the fungal membrane by inserting into the lipid bilayers and creating pores that disrupt plasma membrane integrity, allowing small molecules to diffuse across the membrane, resulting in cell death [24]. Nystatin and amphotericin B belong to this group. Nystatin has a spectrum of activity slightly narrower than that of amphotericin B but is active against a number of species yeasts and molds [25]. The topical use of nystatin is considered the most common route of administration and plays an important role in the prophylaxis of oral and systemic candidiasis in full-term and premature newborns, infants, and immunocompromised patients [26,27]. Amphotericin B is still considered the gold standard in the treatment of most fungal infections, especially severe invasive infections [28]. However, amphotericin is toxic to mammalian cells, particularly causing nephrotoxicity. To overcome its toxicity, a variety of reformulated versions has been introduced. Lipid formulations of amphotericin B are better tolerated than amphotericin B deoxycolate [29]. Although having broad-spectrum activity against most fungi, lipid formulations are very expensive, limiting the use to second-line or salvage therapy.

Resistance to amphotericin B is quite unusual and most often results from mutations in the *ERG3* gene (which encodes a C-5 sterol desaturase, an enzyme involved in ergosterol biosynthesis), which lower the concentration of ergosterol in the fungal membrane [30]. Resistance to amphotericin B may also be mediated by increasing catalase activity, with decreasing susceptibility to oxidative damage [31]. Among *C. albicans* isolates, amphotericin resistance is still very rare [32,33,34].

### 2.2. Pyrimidine Analogues

5-Flucytosine is the only representative of this class of antifungals. It acts through conversion to 5-fluorouracil by a cytosine deaminase, which is incorporated into DNA and RNA, inhibiting cellular function and division [24]. Since most filamentous fungi lack cytosine deaminase, the spectrum of flucytosine is restricted to pathogenic yeasts. 5-Flucytosine should be used in combination with other antifungal agents namely amphotericin B, rather than in monotherapy, because resistance develops at high frequency [24]. Resistance among *Candida* correlates with mutations in the enzyme uracil phosphoribosyltransferase (Fur1p) that turns, unable the conversion of 5-fluorouracil to 5-fluorouridine monophosphate [35].

### 2.3. Triazoles

Triazoles are the largest class of antifungal drugs in clinical use and have been deployed for approximately three decades. They are heterocyclic synthetic compounds that inhibit the fungal cytochrome P450 14α-lanosterol demethylase, encoded by the *ERG11* gene (also known as *CYP51*) which catalyzes the late step of ergosterol biosynthesis. These drugs bind through a nitrogen group in their five-membered azole ring to the heme group in the target protein and block demethylation of the C-14 of lanosterol, leading to the substitution of methylated sterols in the membrane. Inhibition of this enzyme results in decreased membrane ergosterol content and accumulation of toxic methylated intermediates, with resultant disruption of fungal cell membrane function, growth inhibition, and, in some cases, cell death [35,36,37]. Triazole antifungal activity is generally fungistatic against *Candida* spp., but fungicidal against *Aspergillus*.

The triazoles include fluconazole, itraconazole, voriconazole, posaconazole, and isavuconazole. Given its excellent safety and low cost profile and the proven efficacy for the treatment of invasive candidiasis, fluconazole remains one of the most commonly used antifungal agents [38]. Voriconazole is a second-generation triazole that is active against all *Candida* species and has a broad spectrum of activity and, like itraconazole, is also fungicidal against some isolates of filamentous fungi [39]. Posaconazole differs in structure from the compact triazoles (fluconazole and voriconazole) in part by its extended side chain (a feature held in common with itraconazole); however, it displays a dioxolane ring altered to a tetrahydrofuran [36,40]. The structural differences between the azoles might seem small, but they dictate its antifungal potency and spectrum, bioavailability, drug interaction, and toxic potential. Posaconazole is currently only available as oral formulation, and it must be taken with food or a nutritional supplement, somewhat limiting its usefulness. The drug is well tolerated, with an overall safety profile comparable to that of fluconazole [40]. Isavuconazole is an expanded-spectrum triazole with excellent in vitro activity against yeasts and molds [41,42,43]. It is consider, as well as voriconazole, a gold standard drug for invasive aspergillosis in patients with underlying hematological malignancies [44].

The major mechanism responsible for high level of azole resistance is the overexpression of cell membrane efflux pumps [45,46]. Two classes of pumps are responsible for lowering the accumulation of azoles inside the yeast cell by actively translocating compounds across cell membrane: ABC pumps and the major facilitator (MF) transporters [1,11,13,47,48,49,50,51,52,53,54,55].

The ABC pumps, also called ATP-binding cassette, use the hydrolysis of ATP as an energy source. They have low specificity since they accept as substrates azoles but also a wide range of compounds [56]. The most frequently encountered triazole resistance mechanism among clinical isolates of *C. albicans* is the upregulation or overexpression of mainly *CDR*1 and *CDR*2 genes [57,58,59,60]. Their expression is regulated by the zinc finger transcription factor Tac1, which binds to the drug response element (DRE) found in their promoter [61]. Loss of heterozygosity of specific genomic regions, the increase of chromosome copy number or chromosome aneuploidies have been associated with azole resistance [62,63,64]. *CDR* expression is increase by gain-of-function mutations in Tac1p, with high level of fluconazole resistance occurring when this mutation is couple with loss of heterozygosity [62]. Interestingly, *TAC1* is located in the left arm of chromosome 5 (Chr5), the same chromosome where mating-type-locus (*MTL*) is located [65]. *C. albicans* exhibits two MTL alleles, *MTLa* and *MTLα*, and the loss of heterozigoty at MTL locus is frequently associated with homozygosity at the *TAC1* and *ERG11* loci. Some authors described this homozygosity to be related to antifungal resistance [62,63,66,67]. However, others demonstrated homozygosity at MTL to be infrequent among clinical isolates and that it does not influence directly antifungal resistance [64,68,69].

The second main class of multidrug transporters involved in azole resistance is the MF class. *MDR1* gene is involved specifically in resistance to fluconazole rather than to other azoles and uses the proton motive force of the membrane as an energy source [70,71]. The multidrug resistant regulator, Mrr1, is the transcription factor that controls the expression and is upregulated with *MDR1* in drug resistant clinical isolates [55,71]. The gain-of-function in the transcription factor Mrr1p, followed by loss of heterozygosity, represents the main cause of *MDR1* overexpression in fluconazole resistant *C. albicans* strains [72].

Another mechanism that operates in order to overcome the effect of the drug in the yeast cell is the alteration of the target enzyme Erg11, where at least 12 mutations have been associated with azole resistance, avoiding the binding of the drug to the target [73,74]. In *C. albicans*, point mutations in *ERG11* resulting in amino acid substitutions (G464S) have been associated with fluconazole resistance [75]. Upregulation of *ERG11* due to the amplification of the copy number of the gene is another approach used by the fungal cell in order to overcome antifungal action [76]. *ERG11* overexpression can be achieved through mutations in the transcription factor Upc2 in *C. albicans* [77,78,79]. This transcription factor binds to the azole-responsive enhancer element (ARE) in the *ERG11* promoter [80]. Upc2 also binds to two distinct regions on its own promoter to autoregulate expression during azole exposure [81].

### 2.4. Echinocandins

Echinocandins are at present considered the first-line therapy for *Candida* invasive infections [17]. These compounds are fungicidal in vitro against yeasts. Three agents are presently available for clinical use: caspofungin, micafungin, and anidulafungin. They inhibit β-(1,3) glucan synthase, an enzyme complex that is located in the plasma membrane of fungal cells [24,82,83,84]. This enzyme has a minimum of two subunits, Fks1, the catalytic subunit, and Rho, a GTP-binding protein that regulate the activity of the glucan synthase [84]. They are responsible for the production of β-(1,3) glucan which is essential for fungi as it represents one of the major components of the fungal cell wall [84]. The safety profile of echinocandins is excellent, with few reported adverse events and drug interactions. Despite their considerably greater cost, echinocandins are replacing fluconazole as the antifungal of choice in the Intensive Care Unit (ICU) setting [38].

Recent studies have shown that echinocandins are efficacious and safe, supporting the recommendation as a first-line option in case of bloodstream infections [17].

Echinocandin resistance in *Candida* spp. has been attributed to mutations in the *FKS1* gene, the catalytic subunit of β-(1,3)-glucan synthase, and in a lesser extent in *FKS2*, resulting in amino acid substitutions in conserved regions hot spot 1 (HS1) and hot spot 2 (HS2) [15]. These mutations turn the mutant enzyme 50- to 3000-fold less sensitive to the drug, being amino acid changes at Ser645 as the most pronounced resistant phenotype [85,86]. Acquired mutations in *FKS1* and *FKS2* genes have been predominantly found at position 645 (Serine), S645F (serine to phenylalanine), S645P (serine to proline), and S645Y (serine to tyrosine), and have now been identified in a wide range of *Candida* clinical isolates [84,85,87]. In *C. albicans* mutations encoding an *FKS1,* HS1 alteration S645P, S629P, S654P, F641S, and F641I and in *FKS1*, HS2 alteration R1361G are common [88,89]. Nevertheless, the prevalence of Fks mutations in geographically distinct clinical isolates remains low [86]. A survey of *C. albicans* and *C. glabrata* bloodstream isolates in Switzerland showed that echinocandin resistance remained at a low level despite a significant increase in echinocandin use and was mainly associated with individual pre-echinocandin exposure of prolonged duration [89]. Hot spot mutations are more likely to confer resistance to caspofungin than to anidulafungin or micafungin. Such fact suggests that caspofungin could be less potent than the other two drugs [86,90]. However, these differences in echinocandin potency are abolished in the presence of human serum and therefore cross-resistance is likely to occur in vivo [91,92].

## 3. Factors Contributing to *Candida albicans* Clinical Resistance

For clinicians, the three main issues of concern about antifungal resistance are: how commonly does it occurs, how easy it is to induce through inappropriate usage of antifungal agents, and how often does it result in treatment failure. Clinical resistance may be defined as the persistence or progression of an infection despite appropriate antifungal therapy with an in vitro susceptibility of the organism [23,93]. Given this definition, it is reasonable to affirm that clinical resistance and antifungal tolerance are intrinsically related. The ability of the pathogen to tolerate drug concentrations above the minimal inhibitory concentration (MIC) values is defined as antifungal tolerance, which may promote the acquisition of antifungal resistance [93].

**Figure 1:**
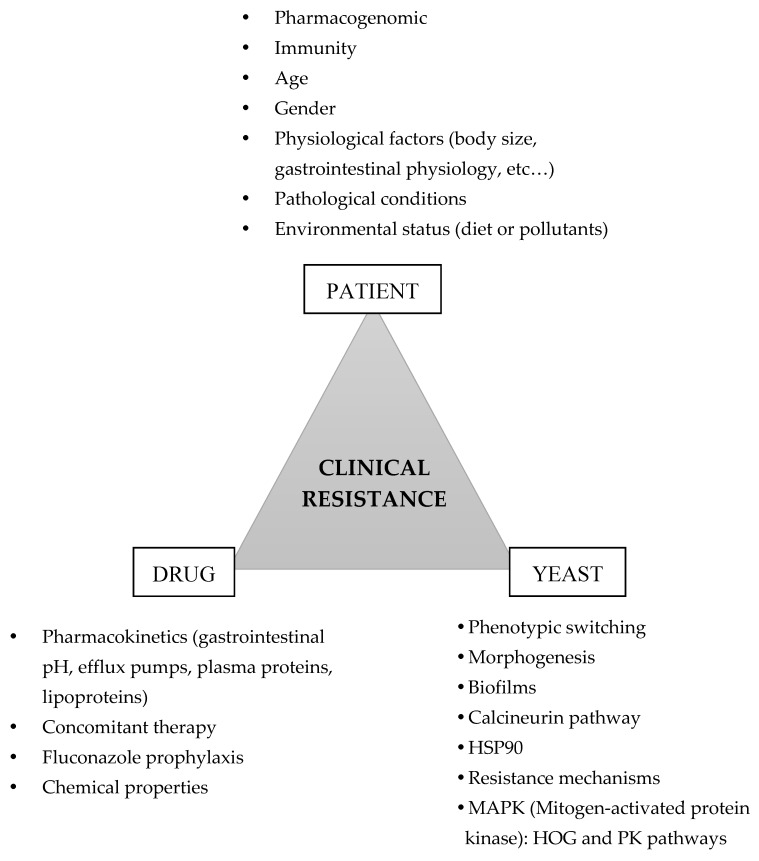
Risk factors that contribute to clinical resistance. Information was collected from the following references: [5,13,47,54,76,95,100,103,122,126,141,142,143,144,145,146].

*C. albicans* has been the subject of extensive research in order to unveil the mechanisms governing fungal virulence and drug tolerance. Many factors may contribute to clinical resistance and to the discrepancy between the laboratory susceptibility pattern and the clinical outcome (Figure 1). The antifungal efficacy lives in the Bermuda triangle that encompasses patient, drug, and yeast factors that ultimately are responsible for a poor clinical outcome. Patient pharmacogenomics, which can influence drug absorption, distribution and metabolism, its immunological status, and the underlying disease are additional important factors to be considered when managing individual patients [94]. Clinical resistance mechanism involving the ability of *C. albicans* to better tolerate and survive high concentrations of antifungal drugs will be the focus of this section.

### 3.1. The Tolerance Pathways

Under stressing conditions like antifungal exposure, *Candida* cells may exploit several cellular responses, such as development of mutations, overexpression of multidrug efflux pumps, modulation of the cAMP protein kinase A (PKA) or Ca^2+^-calmodulin-calcineurin pathways [95].

The stress responses mediating triazole resistance most often involve the cyclic AMP (cAMP)-protein kinase A (PKA) signaling pathway [96,97]. The cAMP-PKA pathway in *C. albicans* is likely required to facilitate the recovery process and resume growth after various stress conditions, like fluconazole exposure. CDC35, encoding the adenyl cyclase enzyme, and CAP, the cAMP-associated protein, are involved in azole tolerance. Disruption of either gene results in hypersusceptibility to azoles that can be partially reverse by the addition of cAMP [96].

Calcineurin is a heterodimeric phosphatase that is involved in calcium-dependent signaling and regulation of diverse cellular processes [35,98,99]. In *C. albicans*, it is involved in virulence, membrane stress response, and is required for the survival in the presence of antifungal drugs, specifically fluconazole [100]. Jia et al. found that calcium-activated-calcineurin, through its target Rta2p and the transcriptional factor Crz1, dramatically reduced the efficacy of fluconazole against *C. albicans*, both in vitro and in vivo [101].

The molecular chaperone heat shock protein 90 (Hsp90) stabilizes calcineurin, enabling calcineurin-dependent stress responses that are required to survive the exposure to fluconazole and echinocandins in *C. albicans* [102]. Cell wall integrity signaling mediated via protein kinase C (PKC), the protein phosphatase calcineurin, and Hsp90, is very important in enabling echinocandin drug tolerance and compensatory mechanisms such as upregulation of chitin synthesis [102]. Echinocandin treatment may trigger cell wall salvage mechanisms producing physiological alterations that decrease the susceptibility to these antifungal agents [103]. The inhibition of the β-(1,3)-glucan synthesis leads to a compensatory increase in chitin synthesis mediated by the PKC cell wall integrity MAP kinase, Ca^2+^-calcineurin and high osmolarity glycerol response (HOG) signaling pathways [104]. Such an increase in chitin content is responsible for the paradoxical growth (PG) or “eagle effect” and occurs most frequently with caspofungin than with anidulafungin and micafungin, most frequently at concentrations well above the MIC level or of sub-MIC level [103,105,106,107,108]. It was also demonstrated that high-chitin *C. albicans* cells are less susceptible to caspofungin [109]. After two hours of caspofungin exposure, chitin content increase significantly, especially in *C. parapsilosis*, *C. tropicalis*, and *C. albicans*, strains that showed PG in microdilution assays [110]. Although some authors state that the PG is eliminated by human serum thus being unlikely to occur in vivo, others have shown that the effect can in fact occur in vivo after exposure to echinocandin treatment [111,112].

A recent study implicated the *C. albicans* transcription factor Cas5, a key transcriptional regulator of cell wall stress response, in governing echinocandin tolerance and an attractive target for antifungal development [113].

Yang and co-workers found mechanisms of caspofungin tolerance in *C. albicans* that may be involved in earlier tolerance development, which involve rearrangements of chromosome 5 (Ch5) [114,115]. Changes of expression of three genes residing on the right arm of Ch5: *CHT2*, implicated in cell remodeling, and *CNB1* and *MID1*, which belong to the calcineurin stress response-signaling pathway were found [115]. Multiple genes may be regulated and involved in order to increase the amount of chitin like downregulation of *CHT2*, *PGA4*, and *CSU51* on the monosomic Ch5, as well as upregulation of *CHS2* and *CHS3* for the chitin synthases encoded outside Ch5 [116].

For the immune system of infected hosts, both chitin and β-glucan act as pathogen-associated molecular patterns (PAMPs), especially β-glucans that are expose on the cell surface and its recognition is mediated by Dectin-1 [117,118]. Echinocandin promotes the efficiency of phagocyte killing, as the inhibition of β-glucan synthase results in a pathogen that is more recognizable by host cells [118]. Thus, this will increase the dectin-1-mediated inflammatory response of macrophages to *C. albicans* because of the exposure of the normally concealed branched glucan polymers [117,118]. However, the inhibition of glucan production in the cell wall may trigger the salvage pathway and subsequent increase of chitin content, influencing the dectin-1 receptor recognition, and generating a decrease in inflammatory response [119]. *C. albicans* cells with high chitin content stimulated a lower level of cytokine response than the ones expressing normal chitin levels [118].

### 3.2. Cell Plasticity

The success of *C. albicans* as a pathogen depends largely on its ability to generate diversity not only at the genetic level but also at the morphological and physiological level [120]. *C. albicans* is considered pleomorphic due to its ability to switch from yeast to hyphal or pseudohyphal form [120,121]. Morphogenic changes are coupled to biofilm formation, which plays an important role in virulence (Figure 1). *C. albicans* can produce biofilm on medical implants, like indwelling vascular catheters, and its formation acts as a physical barrier, protecting the underlying cells of antifungal drugs, hence lowering the available drug concentration [122,123]. *Candida* biofilm infections can have devastating consequences, progressing to bloodstream invasive infections since cells are usually resistant to antifungal drugs and to the host immune system. Biofilm cell development follows closely an intricate gene regulatory network genes and complex transcriptional factors like Efg1, Tec1, Bcr1, Brg1, Ndt80, and Rob1, which are involved in cell surface regulation, hyphal formation, and development and virulence expression [124,125].

*Candida* biofilm cell communities strike back antifungal action through multifactorial mechanisms [126,127,128]. Efflux pumps such as the ATP binding cassette transporters (*CDR1* and *CDR2*) and major facilitator transporter (*MDR1*) are involved in azole resistance by *C. albicans* biofilm, especially at the early stages of its formation rather than in mature biolfilms [127,129]. The extracellular matrix of biofilm, a polymeric material that promotes adherence and protects cells from hostile environments, is also a major contributor to antifungal resistance in *C. albicans*. Azoles and conventional amphotericin B are ineffective against *C. albicans* biofilms [130,131]. In contrast, liposomal amphotericin B and echinocandins have showed to exhibit antifungal activity upon *C. albicans* biofilms [131,132,133]. Since the main mechanism of action is to inhibit β-(1,3)-glucan synthesis, echinocandins are able to abolish the excess of extracellular matrix production, turning the antifungal effect more effective.

The mitochondrial respiratory pathway can regulate the metabolic behavior contributing to fitness and flexibility of *Candida* organisms in response to external challenges [134,135,136]. The existence of an alternative respiratory pathway, alternative oxidase (AOX), present in some *Candida* species, especially *C. albicans* has been implicated in reduced susceptibility to azoles and resistance to oxidative stress [137,138]. Although the inhibition of this alternative respiratory pathway might seem an attractive strategy to combat *Candida* infections, some authors state that this inhibition may result in different outcomes in turns of other drug susceptibilities, in which the link between mitochondrial respiration and cell wall regulation may vary among species [139,140].

### 3.3. Effect of Concomitant Therapy

Antibacterial drugs administration in high-risk patients is one of the major factors that contributes to the development of candidiasis. They cause an imbalance in the fungal microbiome, increasing colonization and proliferation of yeasts [147]. Concomitant medications administered to patients can influence the pharmacodynamics of the antifungals [19,146]. Fluoroquinolones antagonize fluconazole activity against *C. albicans* strains, whether rifampicin can induce the expression of MDR1 pumps [145,146]. While the effect of other medications, some of them lifesaving in the case of critical care patients remains to be elucidated, it has been demonstrated in vitro that albumin and propofol impair the antifungal susceptibility profile [19,20,21].

## 4. Strategies to Overcome Antifungal Resistance and Tolerance

The knowledge encompassing the mechanism of antifungal resistance brought by the genomic era supports the development of novel therapeutic strategies in order to bypass antifungal drug resistance. The principal cell mechanism of antifungal resistance is the active transport of drugs out of the cell by efflux pumps, a mechanism expressed not only by yeasts but also by human cells [52,53,54,55,59,94]. The main strategy to reduce efflux impact involves the maintenance of a high antifungal concentration inside the cell, at its site of action. The simplest approach would be the use of antifungals that are not substrate of efflux pumps, like amphotericin B or echinocandins, which given its respective hydrophobicity and size, do not interact with the efflux pump [83,148].

The second approach would be the development of inhibitors or chemosensitizers of efflux, impairing the target, activity, by blocking access to the binding site, or even the efflux pump transcription.

In humans, one of the factors that is responsible for the failure of cancer therapy are ATP-dependent drug efflux pumps, such as P-glycoprotein (P-gp) [149]. P-gp substrates such as FK506 [150,151,152] or cyclosporine A (CsA) are immunosuppressors that are able to inhibit efflux [153]. They act similarly in *C. albicans*, inhibiting the calcineurin-mediated azole tolerance by binding to small, abundant, conserved binding proteins called immunophilins. CsA binds to cyclophilin A (Cyp1p) and FK506 to FKBP12, forming protein-drug complexes that inhibit calcineurin [52,100,154]. By inhibiting calcineurin, these compounds act synergistically with azoles [100,155,156]. While FK506 and CsA chemosensitize *C. albicans* cells to azoles, turning the azoles fungicidal, they are simultaneously immunosuppressive drugs, which turns its administration problematic to immunosuppressed patients. Nevertheless, the inhibition of calcineurin-mediated azole tolerance is still a potential therapeutic approach [157]. Non-immunosuppressive analogs could inhibit fungal calcineurin by exploiting structural differences between the human and the fungal targets [157].

Ibuprofen ([2-(4-isobutylphenyl)-propionic acid]) has been described to act synergistically with pyrazinamide [158], fluconazole [59,159,160], and amphotericin B [161] in fungi. In *C. albicans* expressing *CDR* efflux pumps, ibuprofen induced azole intracellular accumulation, changing the resistant phenotype to susceptible [59,160]. In a murine model of systemic infection, ibuprofen acts synergically with fluconazole against a fluconazole-resistant strain, drastically reducing the fungal burden and morbidity [162]. This potent anti-inflammatory, non-steroidal drug might play important role in future therapeutic strategies.

Kaempferol, a flavonoid, exhibited a synergistic effect in *C. albicans* fluconazole resistant strains, by decreasing *CDR*1, *CDR*2, and *MDR*1 expression [163].

Another helpful strategy would be the design of inhibitors that could act indirectly on efflux, de-energizing the ATP or H^+^ dependent transporter, by lowering the cytoplasmic ATP concentration or by depleting the electrochemical potential of the plasma membrane, respectively [56,164]. However, altering ATP and membrane potential could compromise other cellular metabolic activities. Alternatively, the promotion of antifungal uptake could also be a strategy to overcome antifungal resistance due to efflux. Dubikovskaya et al. showed that the inclusion of multiple arginine residues (octaarginine (R8)) in human anticancer drugs enhances the delivery to its intracellular targets [165], an approach that has already been tried in yeasts [164].

Nikkomycin Z is a peptidyl nucleoside that functions as a substrate analogue and inhibits chitin synthase at its catalytic site [166]. To prevent chitin increase, Stevens combined *in vitro* nikkomycin Z with caspofungin and a synergistic effect was obtained [167]. In *A. fumigatus*, it was reported that chitin cell wall content was not affected by nikkomycin Z treatment but was markedly affected by caspofungin plus nikkomycin Z [117,168]. In addition, the combination of the two antifungals led to changes in cell wall chitin and β-glucan content. This synergistic effect was also observed in *Alternaria infectoria* [169]. In the case of *C. albicans* biofilm, the combination of echinocandins with nikkomycin Z demonstrated to be synergic, causing an extended cells death [170,171].

## 5. Conclusions

The last 30 years of medical advances led to a significant increase of life-threatening *Candida* infections. The high incidence and mortality rates associated with invasive candidiasis have remained unchanged for more than a decade despite the advances in the field of antifungal therapy. Such infections could be treated more effectively if faster and more specific diagnostic and therapeutic alternatives were available. Preventive strategies targeting patients with a high-risk profile, the development of new diagnostic tools for early identification of fungal species, its susceptibility profile, also including innate resistant species or those that are more prone to develop multidrug resistance, especially in patients submitted to long-term therapy are of utmost importance.

Given the association between antifungal exposure and the development of resistance, prophylaxis must be selectively restricted to high-risk patients. Apparently, there is no antifungal that is immune to the development of acquired resistance. It is essential that laboratories start performing routinely in vitro susceptibility testing especially in isolates from invasive infections, recovered from patients receiving antifungal prophylaxis and in strains isolated from patients who do not respond promptly to therapy.

Despite the yet low in vitro resistance of *C. albicans* to antifungal drugs, this yeast represents a challenge to clinicians and must not be underestimated.

The medical complexity of patients taken together with the intricate cellular mechanism involved in drug resistance and tolerance makes the pursuit of more effective solutions mandatory.

## Figures and Tables

**Table 1:** Spectrum of activity and mechanisms of action and resistance of the major antifungal agents enrolled in the treatment of invasive candidiasis.

Antifungal Class	Antifungal Drug	Spectrum of Activity	Mechanism(s) of Action	Mechanism(s) of Resistance
*Polyenes*	Amphotericin B	Fungicidal	Polyene molecules links to ergosterol in the fungal membrane by inserting into the lipid bilayers, creating pores that disrupt plasma membrane; oxidative damage.	Mutations in the *ERG3* gene affect ergosterol biosynthesis and content in the fungal membrane is responsible for a decrease access to the drug target; susceptibility to oxidative damage by increasing catalase activity.
*Pyrimidine analogues*	5-Flucytosine	Fungicidal	Inhibition of cellular function and division by incorporating toxic fluorinated pyrimidine antimetabolites into DNA and RNA.	Mutations in the enzyme uracil phosphoribosyltransferase (Fur1p), decreasing the formation of toxic antimetabolites.
*Azoles*	Fluconazole Voriconazole Posaconazole	Fungistatic	Inhibition of the fungal cytochrome P450 14α-lanosterol demethylase and accumulation of toxic methylated intermediates, with resultant disruption of fungal cell membrane function and growth inhibition.	Overexpression of cell membrane efflux pumps, decreasing drug concentration (upregulation or overexpression *CDR* and *MDR* genes); alteration of the target enzyme, decreasing affinity to the binding site (point mutation in *ERG11* gene); upregulation of the target enzyme (overexpression of *ERG11* gene).
*Echinocandins*	Caspofungin Anidulafungin Micafungin	Fungicidal	Inhibition of β-(1,3) glucan synthase, decreasing the production of β-(1,3) glucan, which represents one of the major components of the fungal cell wall.	Point mutations in *FKS1* and *FKS2* genes.
